# Toward Comprehensive Interventions to Improve the Health of Women of Reproductive Age

**DOI:** 10.1371/journal.pntd.0000295

**Published:** 2008-09-17

**Authors:** Jennifer F. Friedman, Luz P. Acosta

**Affiliations:** 1 Center for International Health Research, Rhode Island Hospital and Department of Pediatrics, Brown University, Providence, Rhode Island, United States of America; 2 Department of Immunology, Research Institute of Tropical Medicine, Manila, The Philippines; Case Western Reserve University School of Medicine, United States of America

## Background

In lesser-developed countries (LDCs), the causes of anaemia during pregnancy are multi-factorial, yet much of the aetiological fraction of disease is attributable to a few entities. Iron deficiency is the most common cause of anaemia among pregnant women, resulting from both dietary insufficiency of iron as well as losses through the gastrointestinal tract. These losses are largely due to hookworm infection, but schistosomiasis at higher intensities of infection may also lead to blood loss [1].

The argument for hookworm treatment during pregnancy as proposed by Brooker et al. [2] and the WHO [3] is based largely on expected consequences of hookworm-related iron deficiency anaemia for both mother and newborn. It is beyond the scope of this commentary to address the complex relationship between hemoglobin levels assessed at different stages of pregnancy and peri-natal morbidity [4]. However, the relationship between iron status early in pregnancy and birth outcomes is clearer and more relevant here. It is fairly well established that iron deficiency anaemia present during the first trimester of pregnancy is associated with a 2-fold risk of low birth weight [5]. This risk is much lower among women with non–iron deficiency anaemia during the first trimester, arguing for an important mechanistic role for iron per se, discussed in greater detail in recent reviews [6]. In addition to its effects on birth weight, transfer of iron to the developing fetus is compromised among women with depleted iron stores [7]. Maternal iron deficiency is related to decreased newborn and infant iron stores, as well as increased risk of anaemia during infancy [4]. Given the established relationship between hookworm and iron deficiency, hookworm treatment is likely to positively affect maternal and infant health, though the timing of treatment as well as provision of micro-nutrient supplementation are key factors discussed further below.

## A Systematic Review of Hookworm-Related Anaemia among Pregnant Women

Brooker and colleagues have conducted a timely and informative meta-analysis examining the burden of hookworm among pregnant women. In this meta-analysis, they have included cross-sectional, observational, and randomized controlled trials to estimate the contribution of hookworm infection to maternal anaemia. All studies that provided quantitative data on both hookworm intensity of infection and hemoglobin were included, yielding 19 for inclusion. Overall, hookworm infection during pregnancy was related to a standardized mean difference in hemoglobin of −0.24 g/dL in comparing uninfected to lightly infected women, and −0.57 g/dL in comparing lightly infected to heavily infected women.

## Strengths and Limitations of the Study

The limitations of this study are common to many meta-analyses, whereby the quality of summary estimates are driven largely by the quality of the studies included. This is particularly challenging in the case of meta-analyses of cross-sectional and observational studies where bias and confounding are more likely to play a role than in well-executed randomized controlled trials [8]. Approaches to this issue are to either judge the quality of studies and then exclude based on a particular quality score, or include all studies with careful consideration of the potential for confounding or bias in interpretation of results. The latter approach, taken by the authors, is potentially problematic in this study given that a host of factors related to poverty may be related to both hookworm and anaemia, thus confounding this relationship. These potential confounders include an iron-deficient diet, access to iron supplementation during pregnancy, schistosomiasis, HIV, and malaria infections. If diseases of poverty related to both hookworm infection and anemia are not adjusted for in analyses, this variance may be wrongly attributed to hookworm, overestimating its effect.

Though Brooker and colleagues did not exclude studies based on quality, the summary of designs in Table 1 is fairly reassuring. Many studies from malaria- and schistosomiasis-endemic regions adjusted for these infections, and six of 13 studies adjusted for a marker of poverty such as socio-economic status (SES) or maternal education. An examination of Figure 1 suggests that studies which did and did not adjust for SES seem to provide similar estimates of hookworm effect, as also supported by results of heterogeneity analyses. Though some residual confounding may have led to a slight overestimate of effect, it is unlikely that this would change the paper's conclusions significantly.

Other limitations relate to use of observational studies to assess changes in hemoglobin during pregnancy with treatment for hookworm infection. This is limited by the expected hemodilution that occurs in healthy pregnancies during the third trimester. These studies likely underestimate the effect of treatment given that most studies evaluated hemoglobin early in pregnancy and then during the third trimester.

## Implications

The treatment trials discussed in this manuscript provide the clearest insight into implications of this meta-analysis. It is beyond the scope of this commentary to fully review those studies, but the implications include the following:

1. The importance of concomitant administration of iron-folate supplementation. Hookworm treatment alone may mitigate ongoing losses during pregnancy but does nothing to replenish depleted iron stores, which place mother and fetus at risk. In a Peruvian study, the addition of mebendazole to iron-folate provided no improvement in maternal hemoglobin during the third trimester compared to iron-folate alone [9]. A second study demonstrated a 0.66 g/dL difference in albendazole-treated (early second trimester) versus untreated women, with markedly increased impact with the addition of iron-folate to 2.0 g/dL compared to control [10].
2. The health of women independent of their status during a specific pregnancy is of paramount importance. Each cycle of pregnancy and lactation is tremendously iron demanding, with iron deficiency anaemia the third leading cause of Disability Adjusted Life Years lost for females 15–44 years of age [11]. It is clear that hookworm treatment plus iron supplementation mitigates iron deficiency and leaves women to face both future pregnancies and activities of daily living healthier.
3. With respect to safety, findings are reassuring; however, the safety analyses conducted using the Peruvian [12] and Sierra Lione [10] data provided no estimates of power attained to capture fairly rare outcomes such as stillbirths and malformations. A separate cross-sectional study conducted in Sri Lanka had 80% power to detect an increased odds of 2.0 or more for birth defects and found no increased risk with mebendazole treatment during the second trimester [13]. Further, of noted birth defects among women who did or did not receive mebendazole, there is no obvious pattern to systems involved.

## Next Steps

Much has been learned in the past two decades with respect to the impact of specific diseases of LDCs and their impact on human pregnancy. Next steps will require scientists with expertise in a range of diseases to evaluate more complete and integrated interventions to improve the health of both women of reproductive age and their newborns. This includes the evaluation of optimal timing of interventions. Although pregnancy remains a favorable time to capture women of childbearing age, an approach whereby women enter pregnancy in good health is ideal given the minimization of risks of interventions to the fetus, the limited period of recovery and benefit possible for interventions during pregnancy, and the greater risk for interactions with interventions that must be given during pregnancy such as therapeutics for malaria and HIV. In addition, the examination of multiple simultaneous interventions during pregnancy will be of increasing importance as more drugs become available, most of which have been examined in isolation. It is possible that drug combinations confer a greater than additive risk of side effects. Further need to simultaneously assess interventions is supported by a recent example that raised the specter that iron supplementation during pregnancy may increase malaria risk [14]. These concerns notwithstanding, there is great potential to improve the health of pregnant women in the coming decades if industrialized nations provide much needed support, scientists from a range of backgrounds collaborate to evaluate multiple concurrent interventions, and women's socio-political status independent of their role as mothers is improved.
